# The microRNA-218~Survivin axis regulates migration, invasion, and lymph node metastasis in cervical cancer

**DOI:** 10.18632/oncotarget.2836

**Published:** 2014-11-25

**Authors:** Ryunosuke Kogo, Christine How, Naz Chaudary, Jeff Bruce, Wei Shi, Richard P. Hill, Payam Zahedi, Kenneth W. Yip, Fei-Fei Liu

**Affiliations:** ^1^ Ontario Cancer Institute, University Health Network (UHN), Toronto, Ontario, Canada; ^2^ Department of Medical Biophysics, University of Toronto, Toronto, Canada; ^3^ Department of Radiation Oncology, University of Toronto, Toronto, Canada; ^4^ Department of Radiation Oncology, Princess Margaret Cancer Centre, UHN, Toronto, Canada

**Keywords:** miR-218, survivin, cervical cancer, migration, invasion

## Abstract

Cervical cancer is the third most common cancer in women worldwide. In the present study, global microRNA profiling for 79 cervical cancer patient samples led to the identification of miR-218 down-regulation in cervical cancer tissues compared to normal cervical tissues. Lower miR-218 expression was associated significantly with worse overall survival (OS), disease-free survival (DFS), and pelvic/aortic lymph node recurrence. *In vitro*, miR-218 over-expression decreased clonogenicity, migration, and invasion. Survivin (BIRC5) was subsequently identified as an important cervical cancer target of miR-218 using *in silico* prediction, mRNA profiling, and quantitative real-time PCR (qRT-PCR). Concordant with miR-218 over-expression, survivin knockdown by siRNA decreased clonogenicity, migration, and invasion. YM155, a small molecule survivin inhibitor, significantly suppressed tumor growth and lymph node metastasis *in vivo*. Our findings demonstrate that the miR-218~survivin axis inhibits cervical cancer progression by regulating clonogenicity, migration, and invasion, and suggest that the inhibition of survivin could be a potential therapeutic strategy to improve outcome in this disease.

## INTRODUCTION

Cervical cancer is the third most common cancer in women globally [1]. In patients with locally advanced cervical cancer, cisplatin-based concurrent chemoradiotherapy can improve overall survival, progression free survival, and recurrence rates [2-4]; however, the 5-year survival rates for stage III and IV patients remains at less than 40% [5]. Moreover, approximately 30% of patients experience lymph node recurrence and distant metastasis after primary treatment [6]. The ability to prevent lymph node and distant metastasis remains an important yet unresolved therapeutic goal for these patients. Recent molecularly targeted therapeutics have shown potential in decreasing metastasis and improving survival for several human malignancies [7]; however, no proven drugs yet exist for cervical cancer.

MicroRNAs are small, non-coding RNAs that post-transcriptionally down-regulate the expression of multiple target genes [8]. MicroRNA dysregulation occurs in numerous human malignancies and is associated with altered malignant potential; affecting survival, proliferation, apoptosis, and invasion [9]. Recently, global profiling studies have enabled microRNA-based stratifications of cancer types and patient outcomes [10, 11]. However, the function and target genes of many microRNAs remain to be elucidated; this characterization is fundamentally necessary in order to acquire a deeper understanding of cancer progression.

MicroRNA-218 (miR-218) down-regulation has been reported in several human malignancies, including head and neck squamous cell carcinoma (HNSCC), non-small cell lung cancer (NSCLC), pancreatic ductal adenocarcinoma (PDAC), and gastric cancer [12-15]. In cervical cancer, lower miR-218 serum levels have been described to be associated with tumor invasion [16]. Thus, miR-218 is a clinically important and interesting microRNA for investigation.

In the current study, new miR-218-related associations were identified in clinically annotated cervical cancer samples. Furthermore, the cellular and molecular functions of miR-218 and one of its key targets, survivin (BIRC5), were elucidated. Lastly, we validated these observations *in vitro* and *in vivo* using a small molecule survivin suppressant (YM155), and provide data in support of targeting the miR-218~survivin axis in cancer therapy and preventing metastasis.

## RESULTS

### miR-218 down-regulation was associated with reduced survival in cervical cancer patients

**Figure 1:**
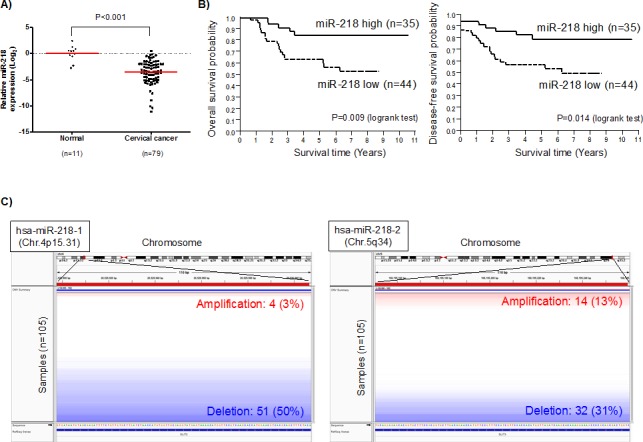
miR-218 down-regulation is associated with poor survival in cervical cancer patients A) miR-218 expression in 79 cervical cancer patient samples and 11 normal cervix epithelial samples. miR-218 expression (log_2_) was measured using Taqman Low Density Array (TLDA) Human MicroRNA A Arrays V2.0 for 79 cervical cancer tissues and 11 normal cervix tissues. B) Kaplan-Meier analysis of overall (*left*) and disease-free survival (*right*) in 79 cervical cancer patients. *Solid line*: miR-218 high expression group (n=35); *dotted line*: miR-218 low expression group (n=44). C) Genomic alteration of miR-218 loci (*left*: hsa-miR-218-1, chromosome 4p15.31; *right*: hsa-miR-218-2, chromosome 5q34) using copy number data from 105 cervical squamous cell carcinoma samples generated by TCGA using SNP 6.0 arrays. Genomic alteration was visualized using the IGV (Integrative Genomic Viewer, Broad Institute). *Blue* represents genomic deletion and *red* represents genomic amplification.

Analysis of Taqman Low Density Array (TLDA) data determined that expression of miR-218 was significantly reduced in 79 cervical cancer tissues compared to 11 normal cervix tissues (P<0.001; Figure 1A). Further details of this study have been described in How *et al.* [17]*;* in brief, these patients have all been treated for cure (radiation and chemotherapy) with a median follow-up time of 6 years. We therefore investigated the association between miR-218 expression with patient survival. Initially, the median miR-218 expression value was utilized to divide the 79 cervical cancer patients into high *vs.* low expression groups (miR-218 high_median_, n=39; miR-218 low_median_, n=40). The miR-218 low expression group experienced a worse overall survival (OS), and disease-free survival (DFS) (OS P=0.074; DFS P=0.079, Figure S1), but the data were of borderline statistical significance. The groups were then re-divided, based on the lowest level of miR-218 expression measured in the normal cervix population. This resulted in 35 patients with high miR-218 expression *vs.* 44 with low miR-218 expression. Using this new cut-off level, the low miR-218 expression group experienced a significantly poorer outcome with regards to both OS and DFS (OS P=0.009; DFS P=0.014; Figure 1B). These data suggest that cervical cancer patients with lower miR-218 expression levels than detected in normal cervical epithelium tissues will experience a poor outcome.

Clinical factors were also analyzed for the miR-218 high *vs.* low expression groups (Table 1). The two groups did not differ in age, tumor size, International Federation of Gynecology and Obstetrics (FIGO) staging, or distant metastasis. Of note however, miR-218 down-regulation was strongly associated with pelvic and para-aortic lymph node recurrence (P=0.032 and P=0.013, respectively), as well as an association with lymph node metastasis at the time of diagnosis (P=0.053).

**Table 1:** miR-218 expression and clinical factors

	miR-218 low (n=44)		miR-218 high (n=35)		
Factors	number	%	number	%	P value
Age (mean ± SD)	49.1 ± 2.1 years		51.7 ± 2.4 years		0.41
Tumor size					
< 5cm	27	61.4	21	60.0	0.9
> 5cm	17	38.6	14	40.0	
FIGO staging					
Stage I	15	34.1	9	25.7	0.42
Stage II, III	29	65.9	26	74.3	
Lymph node metastasis					
Absent	18	40.9	22	62.9	0.053^*^
Present	26	59.1	13	37.1	
Recurrence					
Pelvic LN recurrence					
Absent	30	68.2	31	88.6	0.032^**^
Present	14	31.8	4	11.4	
Para-aortic LN recurrence					
Absent	37	84.1	35	100.0	0.013^**^
Present	7	15.9	0	0.0	
Distant metastasis recurrence					
Absent	36	81.8	32	91.4	0.22
Present	8	18.2	3	8.6	

SD: Standard deviation, LN: Lymph node,

* P<0.1,

** P<0.05.

### Deletion of miR-218 genomic loci in cervical cancer

In other cancers, miR-218 down-regulation is known to occur through promoter hypermethylation or genomic loss [12, 13, 18]. In order to elucidate the mechanism of miR-218 down-regulation in cervical cancer, The Cancer Genome Atlas (TCGA) genomic data (SNP arrays) and epigenetic data (methylation profiles) from 105 cervical squamous cell carcinoma samples were analyzed. Copy number data indicated that most patients' miR-218 genomic loci were deleted (hsa-miR-218-1: 50%, hsa-miR-218-2: 31%, Figure 1C). Methylation and microRNA sequencing data were also analyzed, but no correlations were observed between miR-218 methylation and miR-218 expression level (data not shown). Overall, these data suggest that in cervical cancer, reduced miR-218 expression level is likely related to deletion of the miR-218 loci.

### miR-218 reduced cell survival, migration, and invasion *in vitro*

**Figure 2:**
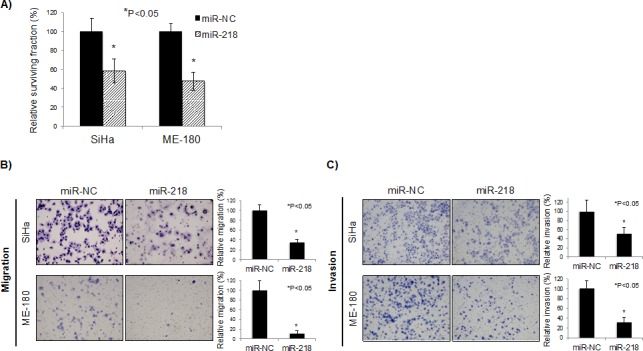
miR-218 reduces survival, migration, and invasion *in vitro* A) Clonogenic assays were performed by seeding SiHa and ME-180 cells transfected with 10 nM each of pre-miR negative control (miR-NC) or pre-miR-218 (miR-218). At 48 hrs post-transfection, cells were re-seeded at low density in 6-well plates. After 10-14 days incubation, colonies were stained and counted. B) Representative image (*left*) and quantification bar graph (*right*) of migrated SiHa and ME-180 cells. Migration assays were performed by seeding SiHa and ME-180 cells transfected with miR-NC or miR-218 (10 nM each) in trans-well chambers. After 48 hrs incubation, the number of migrated cells were stained and counted. C) Representative image (*left*) and quantification bar graph (*right*) of invaded SiHa and ME-180 cells. Invasion assays were performed by seeding SiHa and ME-180 cells transfected with miR-NC or miR-218 (10 nM each) in Matrigel invasion chambers. After 48 hrs incubation, the number of invaded cells were stained and counted. A-C: Bar graphs represent mean ± SEM from triplicates. *P<0.05, miR-NC: pre-miR negative control; miR-218: pre-miR-218.

In order to elucidate the biological significance of miR-218 down-regulation, SiHa and ME-180 cells, which are both human papillomavirus (HPV) positive cervical squamous cell carcinoma lines, were transfected with pre-miR negative control (miR-NC) or pre-miR-218 (miR-218). Forty-eight hours post-transfection, miR-218 was over-expressed by more than 200-fold in SiHa and ME-180 cells (P<0.01, Figure S2). In both cell lines, this over-expression significantly reduced clonogenicity (P<0.05, Figure 2A).

Because miR-218 down-regulation was observed to be associated with lymph node metastasis and recurrence in our patients, we performed migration and invasion assays. Consistent with the clinical data, miR-218 over-expression reduced migration and invasion capacities of both SiHa and ME-180 cells (P<0.05; Figures 2B and 2C).

### Survivin is an important target of miR-218 in cervical cancer cells

**Figure 3:**
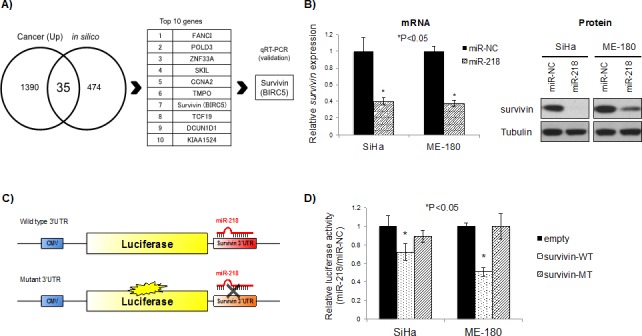
Survivin is a direct target of miR-218 A) Identification of miR-218 targets in cervical cancer. Cancer (Up): mRNA expression greater than 2-fold compared to normal cervix, from GeneChip Human Genome U133 Plus 2.0 Array data for 79 cervical cancer tissues and 11 normal cervix tissues; *in silico*: predicted targets of miR-218 by miRDB (http://mirdb.org/miRDB/). B) mRNA (*left*) and protein (*right*) survivin expression after 48 hrs miR-218 or miR-NC (10 nM each) transfection in SiHa and ME-180 cells. *Survivin* mRNA expression levels were normalized to *GAPDH*. *P<0.05, bars represent mean ± SEM from triplicates. C) Schema of pMIR-REPORT vectors for the luciferase assay. Wild type (*upper*) and/or Mutant (*lower*) survivin 3′UTR were cloned downstream of firefly luciferase gene. miR-218 directly binds to wild type survivin 3′UTR and inhibits luciferase gene expression; on the other hand, miR-218 cannot bind to Mutant 3′UTR and express luciferase gene. D) Relative luciferase activity in SiHa and ME-180 cells after co-transfection with pMIR-REPORT (empty), pMIR-survivin-3′UTR-wild-type (survivin-WT), or pMIR-survivin-3′UTR-mutant (survivin-MT) and miR-NC or miR-218. The values represent the luciferase activity of miR-218 and pMIR-REPORT/miR-NC and pMIR-REPORT. Data were normalized to the luciferase activity of empty pMIR-REPORT transfected cells. *P<0.05, bars represent mean ± SEM from six replicates.

In order to identify biologically relevant miR-218 targets, we first examined *in silico* predicted targets using miRDB (http://mirdb.org/miRDB/) [19, 20]. These data were combined with mRNA array (GeneChip Human Genome U133 Plus 2.0) data generated from the same 79 cervical cancer tissues and 11 normal cervix tissues used for TLDA [17]. At the intersection between the *in silico* predicted targets and mRNAs that were up-regulated by greater than 2 fold were 35 candidate targets (Figure 3A; Table S1). For these candidate targets, their *in silico* prediction scores and expression levels were used to rank the genes independently, then these ranks were summed for a cumulative final rank (Table S1).

The top 10 candidate target genes from Table S1 were then assayed in the SiHa and ME-180 cells using qRT-PCR after 48 hrs of miR-218 transfection, with miR-NC transfection as a control (Figure S3). *Survivin* was the most consistently and significantly reduced target after miR-218 transfection in both cell lines (Figure S3; re-presented in Figure 3B, left panel). Correspondingly, miR-218 over-expression also reduced survivin protein expression (Figure 3B, right panel).

In order to confirm direct targeting and binding between miR-218 and the *survivin* 3′-untranslated region (3′-UTR), we cloned the *survivin* 3′-UTR (which included a miR-218 predicted binding site) into the pMIR-REPORT luciferase vector (Figure 3C). Cells transfected with wild type *survivin* 3′-UTR pMIR-REPORT vector (survivin-WT) showed a significant reduction in luciferase activity in both SiHa and ME-180 cells (P<0.05 relative to empty pMIR-REPORT vector, Figure 3D). These inhibitory effects were not observed with a mutant *survivin* 3′-UTR pMIR-REPORT vector (survivin-MT; containing a mutation in the miR-218 binding site), thereby confirming specific and direct survivin 3′-UTR targeting by miR-218.

### Survivin knockdown reduced survival, migration, and invasion in cervical cancer cells

**Figure 4:**
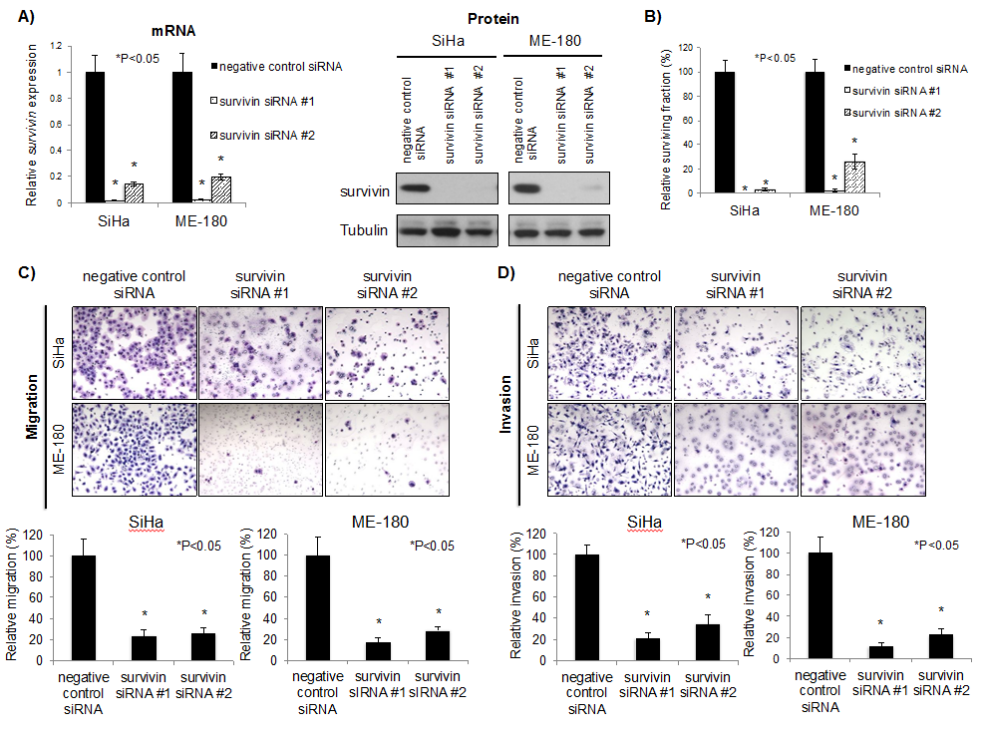
Survivin knockdown reduced survival, migration, and invasion in cervical cancer cells A) mRNA (*left*) and protein (*right*) survivin expression after 48 hrs negative control siRNA or survivin siRNAs (10 nM each) transfection in SiHa and ME-180 cells. *Survivin* mRNA expression levels were normalized to *GAPDH*, relative to cells transfected with negative control siRNA. B) Clonogenic assays were performed by seeding SiHa and ME-180 cells transfected with negative control siRNA or survivin siRNAs (10 nM each). At 48 hrs post-transfection, cells were re-seeded at low density in 6-well plates. After 10-14 days incubation, colonies were stained and counted. C) Representative image (*left*) and quantification bar graph (*right*) of migrated SiHa and ME-180 cells. Migration assays were performed by seeding SiHa and ME-180 cells transfected with negative control siRNA or survivin siRNAs (10 nM each) in trans-well chambers. After 48 hrs incubation, migrated cells were stained and counted. D) Representative image (*left*) and quantification bar graph (*right*) of invaded SiHa and ME-180 cells. Invasion assays were performed by seeding SiHa and ME-180 cells transfected with negative control siRNA or survivin siRNAs (10 nM each) in the Matrigel invasion chambers. After 48 hrs incubation, invaded cells were stained and counted. A-D: *P<0.05, bars represent mean ± SEM from triplicates.

Survivin is the smallest member of the inhibitor of apoptosis (IAP) family, and is mainly associated with the regulation of mitosis and inhibition of apoptosis [21]. Some functions of survivin still remain unknown because survivin interacts with numerous proteins. In order to characterize survivin function in cervical cancer cells, SiHa and ME-180 cells were transfected with survivin siRNAs. Survivin knockdown was confirmed both at the mRNA and protein level (Figure 4A). This down-regulation was accompanied by a significant reduction in clonogenicity compared to control cells transfected with negative control siRNA (P<0.05; Figure 4B). Additionally, survivin knockdown also reduced migration and invasion capacities, phenocopying miR-218 over-expression (P<0.05; Figures 4C and 4D). Overall, these data support the postulate that the miR-218~survivin axis regulates clonogenicity, migration, and invasion in cervical cancer.

### Efficacy of a survivin targeting compound in cervical cancer

In general, survivin is absent in normal adult cells and only expressed in cancer cells; thus, survivin might serve as a useful drug target [22]. Furthermore, if the miR-218~survivin axis regulates cervical cancer clonogenicity, migration, and invasion, then in turn, a survivin suppressant/inhibitor could be therapeutically important in this disease. Although several survivin inhibitors exist [22], we utilized the readily available YM155. This small molecule suppresses survivin transcription and is currently being assessed in several clinical trials for lymphoma, prostate cancer, malignant melanoma, and NSCLC [23].

**Figure 5:**
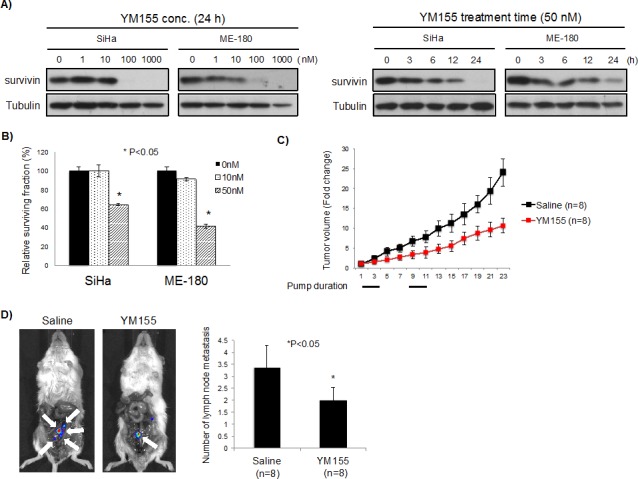
Effect of YM155, a survivin suppressant, *in vitro* and *in vivo* A) YM155 inhibited survivin expression. (*Left*) SiHa and ME-180 cells were treated with YM155 (0, 1, 10, 100, and 1000 nM) for 24 hrs. Survivin expression was analyzed by Western blotting. (*Right*) SiHa and ME-180 cells were treated with YM155 (50 nM) for the indicated period of time. Survivin expression was analyzed by Western blotting. B) Clonogenic assays were performed by seeding SiHa and ME-180 cells treated with YM155 (0, 10, and 50 nM) for 24 h. After 14 days incubation, colonies were stained and counted. *P<0.05, bars represent mean ± SEM from triplicates. C) Luc-ME-180 cell subcutaneous xenografts were treated with YM155 (10mg/kg/day). Saline or YM155 was administered as a 3-day continuous infusion per week for 2 weeks. Tumor volume (mm^3^) was normalized to tumor volume at beginning of treatment (day 1) *P<0.05, bars represent mean (n=8/group) ± SEM. D) Metastatic lymph node analysis. (*Left*) Representative bioluminescence image. White arrows indicate metastatic lymph nodes. (*Right*) Quantification of the number metastatic lymph nodes identified in control (saline) *vs.* YM155 treated mice after 28 days implantation. Saline or YM155 (10 mg/kg/day) was administered as a 3-day continuous infusion per week for 2 weeks after 7 days implantation. Bars represent mean (n=8/groups) ± SEM.

YM155 decreased survivin expression in both a concentration and time dependent manner (Figure 5A). As well, YM155 significantly reduced clonogenicity of SiHa and ME-180 cells, which was comparable to survivin siRNA (Figures 5B and 4B). In order to examine the effects of YM155 against tumor growth and lymph node metastasis in cervical cancer cells, we generated luciferase-expressing ME-180 cells (Luc-ME-180 cells), and evaluated *in vivo* anti-tumor activity. Mice treated with YM155 had significantly reduced tumor growth compared to control mice treated with saline (53% reduction at day 22, P<0.05, Figure 5C). We evaluated intratumoral survivin protein expression at days 0, 3, and 7, and confirmed inhibition of survivin expression during YM155 treatment (compared to saline controls; Figure S4A). Next, the inhibitory effect of YM155 on lymph node metastases was examined using an orthotopic xenograft model of cervical cancer [24]. After 28 days implantation, the number of lymph node metastases in YM155 *vs.* saline treated mice was evaluated using bioluminescence. All metastatic lymph nodes detected by bioluminescence were confirmed using histological analyses (Figure S4B). Importantly, YM155 significantly reduced the number of lymph node metastasis (P<0.05, Figure 5D), without affecting the large primary tumor transplanted into the cervix (data not shown).

## DISCUSSION

The current study demonstrated that miR-218 is significantly down-regulated in human cervix cancer, which in turn, was associated with poor OS and DFS, as well as increased risk of lymph node recurrence. The identified miR-218~survivin axis regulated clonogenicity, migration, and invasion of cervical cancer cells *in vitro*. Furthermore, a small molecule survivin suppressant, YM155 was able to reduce tumor growth and lymph node metastasis *in vivo*.

miR-218 down-regulation has been reported in several cancers [12-15]; it has been shown to inhibit migration and invasion in gastric cancer [15], glioma [25], head & neck [26], renal cell [27], and cervical cancers [28]. Consistent with previous reports, in this current study, we observed that miR-218 inhibited migration, and invasion in cervical cancer cells. Our study further validated and extended upon these findings both clinically, by identifying the association with lymph node involvement at presentation, nodal recurrence, OS, DFS; and experimentally, by elucidating the role of survivin as a downstream mediator of miR-218 under-expression.

The mechanism of miR-218 down-regulation may depend on the type of cancer. To date, promoter hypermethylation in nasopharyngeal carcinoma [12] and genomic deletion in non-small cell lung [18] and bladder cancers [13] have been reported. Our analysis of the data obtained from TCGA SNP arrays suggested that genomic deletion of miR-218 loci is likely a mechanism for miR-218 under-expression in cervical cancer. miR-218 is located in two genomic loci; 4p15.31 (hsa-miR-218-1) and 5q34 (hsa-miR-218-2), which are located within the intronic regions of SLIT2 and SLIT3, respectively. Loss of chromosome 4p has been previously reported in cervical cancer [29], which interestingly, was also noted to be associated with lymph node metastasis [29].

Several studies have identified miR-218 targets, such as ROBO1 [15], IKK-β [25], Laminin-332 [26], Rictor [30], and survivin [12]. We chose to focus only on a single, key target of miR-218 in cervical cancer, identified by combining *in silico* prediction and global mRNA array data in order to evaluate this axis in some depth. Future studies should delve into the relevance of the other targets in similar model systems. For example, bioinformatics-related analyses may be expanded, and survivin-lacking cells may be utilized to compare the contributing effects of survivin *vs*. other targets in miR-218-induced phenotypes. Survivin is an IAP and functions as an oncogene in cancer cells due to its anti-apoptotic properties. The main functions of survivin include inhibition of caspase-dependent apoptosis and caspase-independent cell death, as well as in the regulation of mitosis [21]. However, a complete understanding of all of survivin's functions remains unclear since this protein interacts with a number of other proteins with multi-functional effects [21].

Our data showed that survivin knockdown by siRNA significantly reduced clonogenicity, migration, and invasion in SiHa and ME-180 cells, phenocopying the results of miR-218 over-expression. Survivin is known to promote tumor cell invasion (*in vitro*) and metastasis (*in vivo*), in cooperation with XIAP, another IAP family member [22, 31]. Future work will be required to further elucidate the detailed mechanisms by which survivin affects migration and invasion.

Survivin is typically absent in normal adult cells (except for germ cells), and is highly over-expressed in cancer cells, thereby serving as a drug target. YM155 leads to the repression of *survivin* promoter activity by binding to the transcription factor ILF3 and disrupting the ILF/p54 complex [32]. Phase II clinical trials have been conducted with YM155 alone or as part of combination therapy in prostate cancer, lymphoma, melanoma, and non-small cell lung cancer patients [23]. Our data demonstrated that YM155 significantly reduced survivin expression in a concentration and time-dependent manner, and reduced cell proliferation *in vitro*. We present the first orthotopic metastatic model for YM155 evaluation, and demonstrated that YM155 indeed reduced lymph node metastasis. Primary tumor growth was reduced subcutaneously, but not orthotopically. We speculate that the tumor size (large donor tumors needed to be transplanted in the orthotopic model), and/or the microenvironment might well account for this discrepant observation. Other survivin-inhibiting compounds are currently in development and might be even more effective inhibitors [22]. Nonetheless, we have demonstrated the proof-of-concept that such inhibitors should be further explored in the prevention of metastasis in this disease.

The role of the mir-218~survivin axis in potentially promoting nodal metastasis may suggest a therapeutic opportunity for survivin inhibitors in treating node-positive, locally advanced cancers, and/or cancers with occult nodal involvement. Survivin inhibition may be particularly helpful for adjuvant therapy used to inhibit lymph node metastasis after primary tumor resection. Further work will be required to investigate the use of survivin inhibitors with existing standard treatments (e.g., chemotherapy and/or radiation) for such disease. Moreover, whether this therapeutic opportunity exists in other cancers (e.g., head and neck, lung) remains to be investigated.

In conclusion, the miR-218~survivin axis is pivotal, both on a clinical and basic cellular level, in regulating clonogenicity, migration, and invasion in cervical cancer. Anti-survivin therapy might provide a potentially useful strategy in partially restoring this axis, and thereby improve outcome for patients with cervical cancer.

## METHODS

### Ethics Statement

Written informed consent was obtained from patients according to a protocol approved by the University Health Network (UHN) Research Ethics Board. Animal experiments were performed in strict accordance with the protocols approved by the Animal Care Committee (ACC) of the Ontario Cancer Institute (OCI), UHN.

### Patient Samples and microRNA/mRNA Profiling

Seventy-nine cervical cancer tissues and 11 normal cervix tissues were collected from fresh frozen punch biopsies. Total RNA was isolated using the Total RNA Purification Kit (Norgen Biotek), according to the manufacturer's protocol. Global microRNA and mRNA profiles were analyzed with the TLDA Human MicroRNA A Array V2.0 (Life Technologies), and GeneChip Human Genome U133 Plus 2.0 Array (Affymetrix), respectively.

### Copy Number Analysis of TCGA Data

Level 3 segmented copy number data for 105 cervical squamous cell carcinoma samples generated by TCGA using SNP 6.0 arrays were downloaded from the Broad Firehose website (2012_11_02 stdata Run). Copy number data for miR-218 encoding loci were then visualized using the integrated genome viewer (igv; https://www.broadinstitute.org/software/igv) [33].

### Cell Lines and Transfections

Cervical cancer cell lines, SiHa and ME-180, were obtained from American Type Culture Collection (ATCC) and cultured in α-MEM with 10% Fetal Bovine Serum at 37°C, 5% CO_2_. These cells were authenticated every six months at the Centre for Applied Genomics (Hospital for Sick Children, Toronto, Canada) using the AmpF/STR Identifier PCR Amplification Kit (Applied Biosystems); as well, they were determined to be mycoplasma free every 3 months using the MycoAlert Mycoplasma Detection Kit (Lonza).

SiHa and ME-180 cells were transfected with Lipofectamine RNAiMAX (Life Technologies), according to the manufacturer's protocol. Pre-miR miRNA negative control, pre-miR-218 (Life Technologies), AllStars Negative Control siRNA, and two survivin siRNAs (Qiagen) were transfected at 10 nM.

### Quantitative Real-Time PCR (qRT-PCR) Analysis

Total RNA was isolated using the Total RNA Purification Kit (Norgen Biotek), and miR-218 expression levels were measured using the TaqMan MicroRNA Assays (Life Technologies). The 2^−ΔΔ^ Ct method was used to calculate relative miR-218 expression, using RNU44 as a reference gene.

mRNA expression levels were measured using qRT-PCR; total RNA was reverse-transcribed using SuperScript III Reverse Transcriptase (Life Technologies). QRT-PCR was performed using SYBR Green PCR Master Mix (Life Technologies) with *survivin* specific primers (Table S2). All mRNA expression levels were normalized to *GAPDH* expression.

### Western Blot Analysis

Total protein was extracted using RIPA buffer (50 mM TrisHCl, 150 mM NaCl, 2 mM EDTA, 1% NP-40, 0.1% SDS), then separated using a Novex 4-20% Tris-Glycine Gel (Life Technologies). Proteins were detected using survivin (1:1000 dilution, Novus Biologicals) and α-Tubulin (loading control, 1:40000 dilution, Sigma) antibodies. Specific proteins were detected using SuperSignal West Pico Chemiluminescent Substrate (Thermo Scientific).

### Viability and Clonogenic Assays

Cell viability was examined using the CellTiter 96 Non-Radioactive Cell Proliferation Assay (MTS assay, Promega), according to the manufacturer's protocol. For clonogenic assays, transfected cells were re-seeded 48 hrs post-transfection at low density in 6-well plates. Cells were incubated for 10-14 days, then fixed and stained with 0.2% methylene blue in 50% methanol. The surviving fraction was calculated by comparison with control cells.

### Invasion and Migration Assays

Invasion and migration assays were performed using the BD BioCoat Matrigel Invasion Chambers and Control Inserts (BD Biosciences), respectively. Briefly, cells (1 × 10^5^ cells/well) were seeded with medium containing low serum (1%) in the upper chamber. The lower chamber was filled with medium containing high serum (20%) as a chemoattractant. Cells were incubated for 48 hrs and then membranes were stained using Diff-Quick (Siemens). A light microscope was used to count the number of migrating and invading cells.

### Luciferase microRNA Binding Assay

Wild-type (WT) or mutant (MT) fragments of the *survivin* 3′-untranslated region (3′UTR) containing the predicted miR-218 binding site were amplified by Platinum Taq DNA polymerase (Life Technologies). Primer sequences are listed in Table S2. Amplified PCR products were cloned in the pMIR-REPORT miRNA expression reporter vector (Life Technologies). Cells were co-transfected with 20 nM pre-miR, 100 ng pMIR-REPORT vector (Firefly luciferase), and 50 ng pRL-SV40 vector (Renilla luciferase, Promega) as a reference control using Lipofectamine 2000 (Life Technologies). Luciferase activities were measured using Dual-Glo Luciferase Assay System (Promega) at 24 hrs post-transfection.

### Generation of Luciferase Expressing ME-180 Cells (Luc-ME-180)

Luciferase containing lentivirus (Lenti-Luc) was generated by transient transfection of 293T cells with psPAX2, pCMV-VSVG (Addgene), and CSII-CMV-Bsd-Luciferase plasmids using Lipofectamine 2000 (Life Technologies). At 48 hrs after co-transfection, lentivirus-containing supernatant was collected, and then passed through a 0.45 μm filter. Cultured ME-180 cells were incubated for 48 hrs with Lenti-Luc, then cultured for 2 weeks in the presence of 4.0 μg/mL Blasticidin S (Life Technologies).

### *In Vivo* Experiments

Six to eight week-old severe combined immunodeficient (SCID) female mice were utilized for all experiments. ME-180 or Luc-ME-180 (1 × 10^7^) cells were injected into the left flank or left gastrocnemius muscle subcutaneously or intramuscularly as indicated. Tumor growth was monitored by measuring tumor volume (length × width^2^/2, mm^3^) or tumor plus leg diameter (mm). YM155 treatment commenced once the tumor volume reached 50 mm^3^ or the leg diameter reached 8 mm. YM155 or saline control (mice randomized) was administered subcutaneously as a 3-day per week continuous infusion for 2 weeks using the Alzet Osmotic Pump® (Model 1003D).

For the orthotopic xenograft model, Luc-ME-180 (1 × 10^7^) cells were injected into the left gastrocnemius muscle intramuscularly (donor mice). Once the leg diameter reached 9-12mm, tumors were excised and dissected. The tumors were then cut into 2-3 mm^3^ fragments in α-MEM media and placed on ice. Recipient mice were anesthetized and their uteruses exposed. A small incision was made in the cervix and a tumor fragment was sutured in place using a single 8-0 silk suture. The peritoneal membrane was closed in two layers using 8-0 silk sutures followed by skin closure using wound clips [24].

### Bioluminescence Imaging

Mice were injected with 150 mg/kg D-Luciferin intraperitoneally. After 10 min post-injection, mice were sacrificed and primary tumors were removed. Metastatic lymph nodes with luciferase expression were imaged using an IVIS Spectrum (Xenogen, Caliper Life Sciences). Images were analyzed with Living Image 4.1 Software (Xenogen, Caliper Life Sciences).

### Statistical Analysis

All experiments have been performed at least three independent times, and the data are presented as the mean ± standard error of the mean (SEM). Statistical significance between treatment groups was determined using the Student's t-test or *Χ*^2^ test. Overall survival curves were plotted according to the Kaplan-Meier method, with the log-rank test applied for comparison. Statistical analyses were performed using JMP5 (SAS Institute).

## SUPPLEMENTARY MATERIAL AND FIGURES


