# Supplementary material for: The microRNA-218~Survivin axis regulates migration, invasion, and lymph node metastasis in cervical cancer
Source: Oncotarget. 2014 Nov 25;6(2):1090–100. doi: 10.18632/oncotarget.2836 (PMC4359219; doi:10.18632/oncotarget.2836)
Supplement: Supplementary file 1 [file oncotarget-06-1090-s001.pdf]

The microRNA-218~Survivin axis regulates migration, invasion, and lymph node metastasis in cervical cancer

Supplementary Material

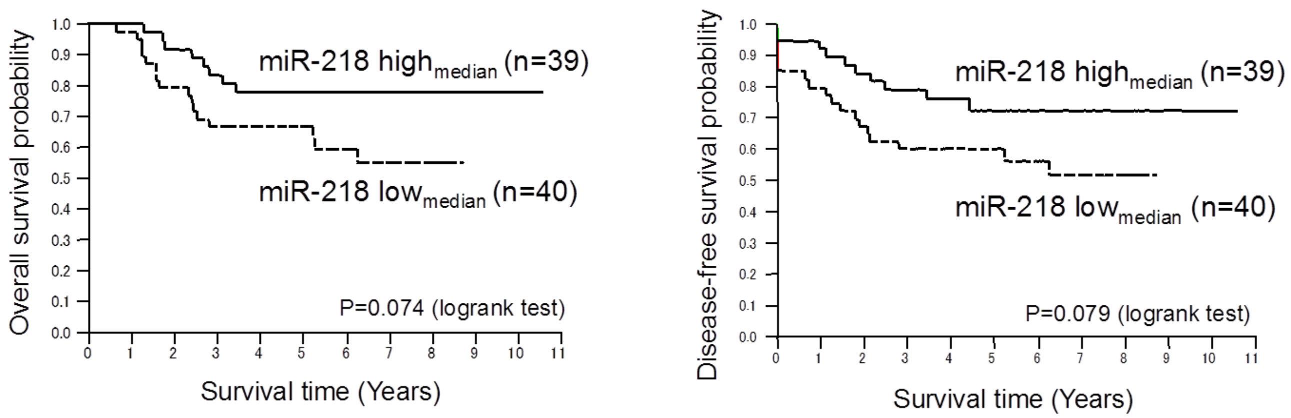

Figure S1: Kaplan-Meier analysis of overall (left) and disease-free (right) survival in 79 patients with cervical cancer. Solid line: patients with higher than median miR-218 expression level (n=39); dotted line: patients with lower than median miR-218 expression (n=40).

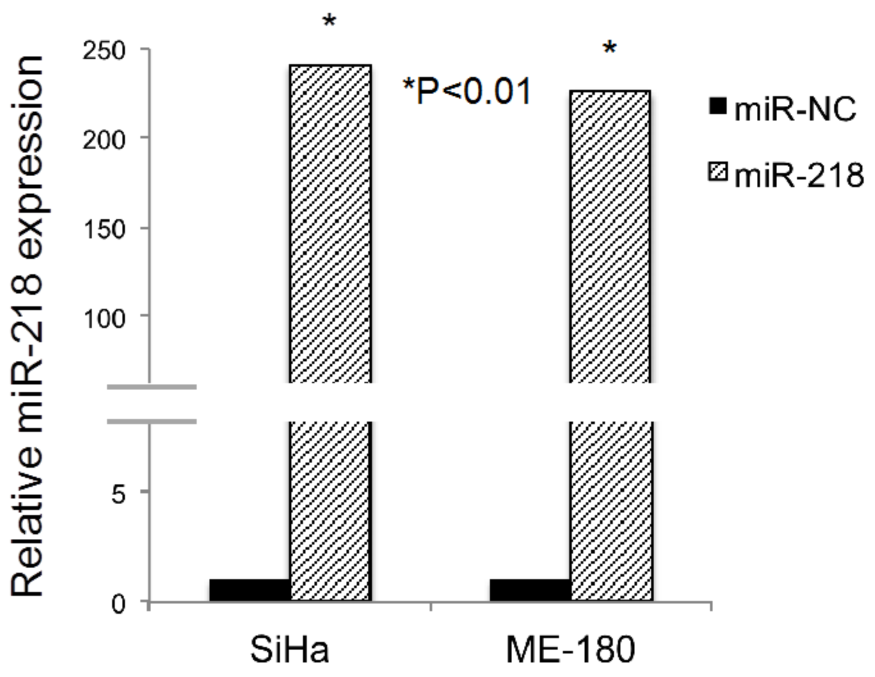

Figure S2: qRT-PCR analysis of miR-218 levels in SiHa and ME-180 cells after 48 h transfection with 10 nM each of pre-miR negative control (miR-NC) or pre-miR-218 (miR-218). miR-218 expression levels were normalized to RNU44 expression, relative to cells transfected with miR-NC.

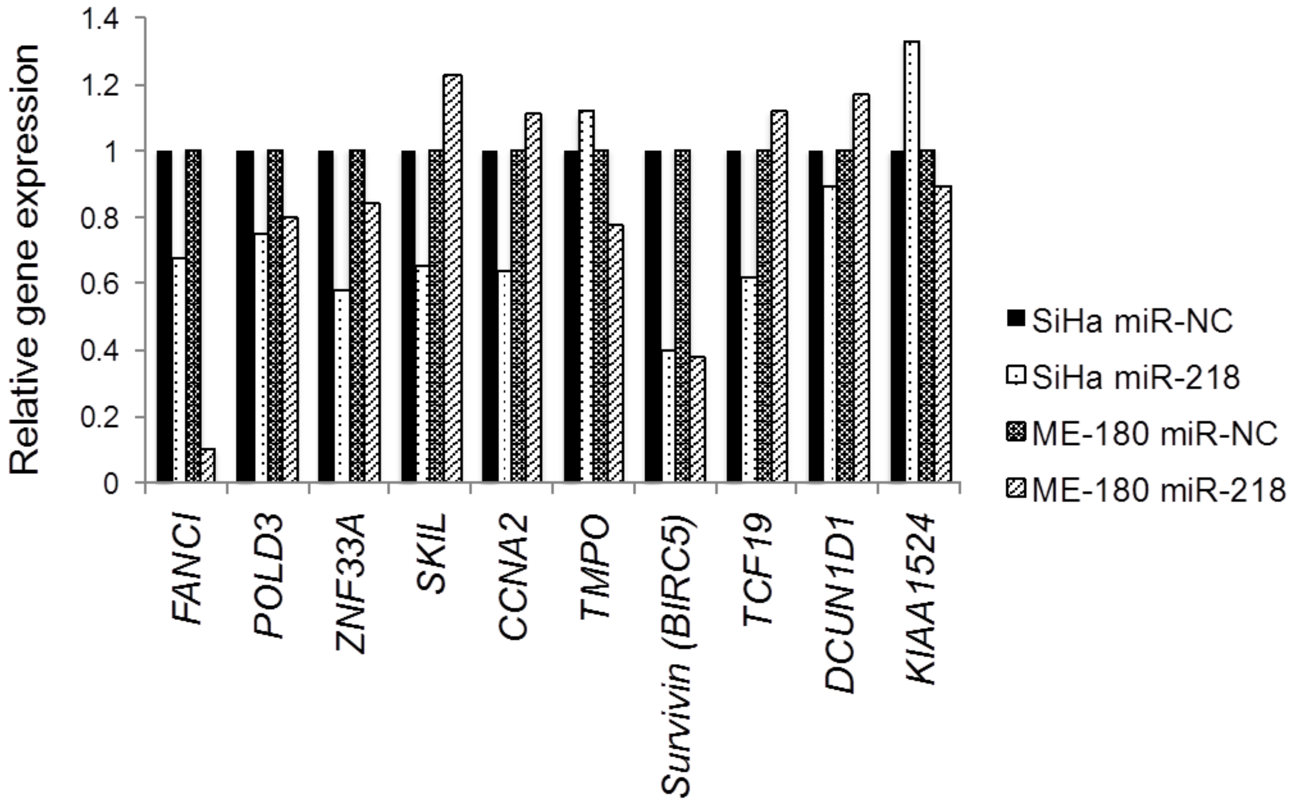

Figure S3: The expression of top 10 candidate genes were assayed using qRT-PCR after 48 hrs miR-218 or miR-NC transfection (10 nM each) in SiHa and ME-180 cells. Gene expression levels were normalized to GAPDH.

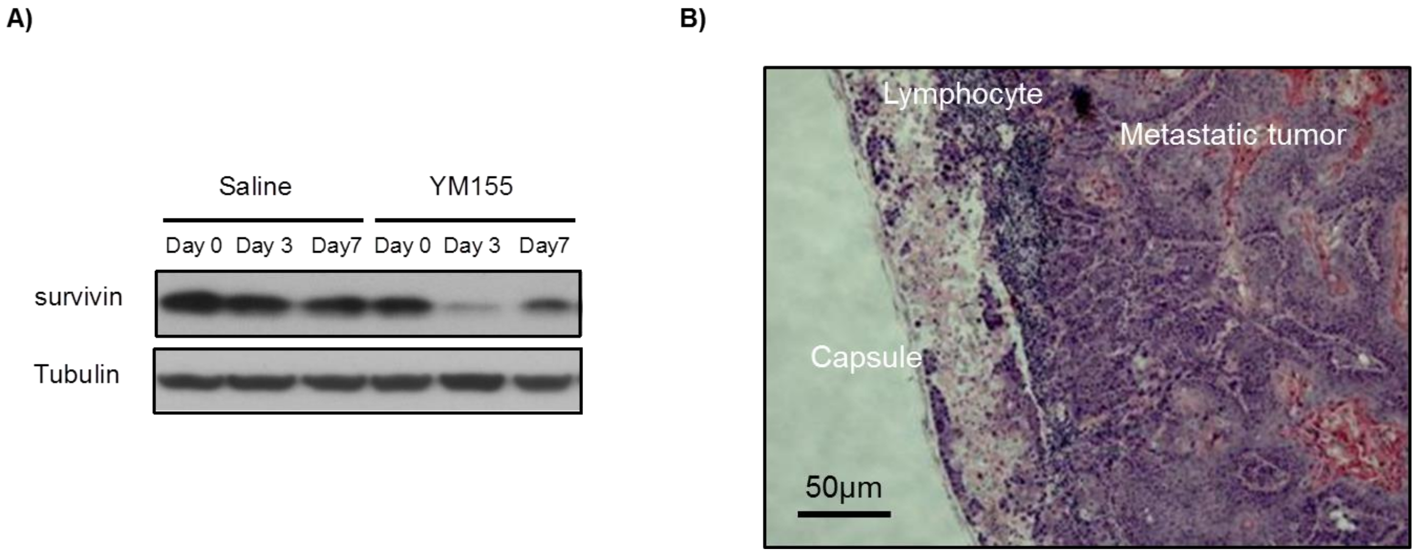

Figure S4: A) Intratumoral survivin expression. After subcutaneous tumor generation, YM155 (10 mg/kg/day) was subcutaneously administered to mice as a 3-day continuous infusion. Subcutaneous tumors were removed at days 0, 3, and 7 after YM155 treatment and intratumoral survivin levels were assessed using Western blotting. B) Representative histology of a metastatic lymph node (H&E staining) demonstrating the capsule of the lymph node, the lymphocyte infiltration, and the metastatic tumor deposit. Scale bar represents 50 µm. These metastatic lymph nodes were detected using bioluminescence.

Table S1: 35 candidate genes for miR-218 targeting

Rank	Gene name	Cancer (Up) rank	*In silico* rank	Sum of cancer (up) + *in silico* rank
**1**	**FANCI**	26	157	183
**2**	**POLD3**	127	218	345
**3**	**ZNF33A**	134	272	406
**4**	**SKIL**	267	155	422
**5**	**CCNA2**	376	80	456
**6**	**TMPO**	174	377	551
**7**	**Survivin (BIRC5)**	361	200	561
**8**	**TCF19**	433	151	584
**9**	**DCUN1D1**	292	303	595
**10**	**KIAA1524**	221	383	604
11	DNA2	111	495	606
12	ANLN	147	509	656
13	VPS13C	610	76	686
14	PHC3	635	116	751
15	DCP2	447	412	859
16	PCNP	570	382	952
17	COL10A1	652	307	959
18	RAD1	874	91	965
19	USP34	869	101	970
20	CPNE8	934	95	1029
21	TRAF5	851	238	1089
22	POLA1	1016	120	1136
23	CDCA7L	1020	120	1140
24	BCAT1	898	255	1153
25	GPAM	948	210	1158
26	HOXD10	1088	93	1181
27	RABGAP1L	1174	12	1186
28	ACPL2	951	277	1228
29	EGLN3	1102	134	1236
30	ZNF678	960	360	1320
31	DCBLD2	1052	283	1335
32	EMR2	1130	391	1521
33	COL1A1	1066	507	1573
34	KIAA1324	1416	185	1601
35	UGT8	1400	337	1737

Table S2: Primer sequence used for qRT-PCR and cloning

Gene	Forward primer	Reverse primer
Survivin (BIRC5)	5'-ACTTGGCCCAGTGTTTCTTCT-3'	5'-ACTTGGCCCAGTGTTTCTTCT-3'
GAPDH	5'-GAGTCAACGGATTTGGTCGT-3'	5'-AATGAAGGGGTCATTGATGG-3'
WT-survivin 3'UTR	5'-GTTGTTACTAGTTGCATGACTTGTGTGT GATGA-3'	5'-GTTGTTAAGCTTAATGACTTAGAA TGGCTTTGTGC-3'
MT-survivin 3'UTR		5'-GTTGTTAAGCTTAATGACTTAGAATGGCT TGTGTTGG AGTTTTAATTGTAGTTTGTG-3'
FANCI	5'-CCACCTTTGGTCTATCAGCTTC-3'	5'-CAACATCCAATAGCTCGTCACC-3'
POLD3	5'-GAGTTCGTCACGGACCAAAAC-3'	5'-GCCAGACACCAAGTAGGTAAC-3'
ZNF33A	5'-AAGATGTGACTGTGGGCTTCA-3'	5'-GATCACCTCTGGTTTGTGAAC-3'
SKIL	5'-CCATCCTGTGGGCTGATTAC-3'	5'-GGCCAATGAGCTTTTAGCAG-3'
CCNA2	5'-GGATGGTAGTTTTGAGTCACCAC-3'	5'-CACGAGGATAGCTCTCATACTGT-3'
TMPO	5'-CCCCTCGGTCCTGACAAAAG-3'	5'-CGCTCTTCGTCACTGGAGAA-3'
TCF19	5'-GGGGCGGTGATCTCTACAC-3'	5'-GGGAGTCGGACATTATTGACCA-3'
DCUN1D1	5'-TCTGTGATGACCTGGCACTC-3'	5'-TGTTTGGAGAACTCGCACTG -3'
KIAA1524	5'-GAACGCCACTCAGCTTTTGC-3'	5'-GAAGCACTTATGTTGGGGTCTT-3'
